# User-Centered Demand Analysis for a Virtual Reality Pelvic Floor Rehabilitation System: Cross-Sectional Study Using the Kano Model

**DOI:** 10.3390/healthcare14111571

**Published:** 2026-06-03

**Authors:** Bing Liu, Xijun Chen, Rui Yang, Mingna Zhang, Qian Xiao

**Affiliations:** 1 Beijing Obstetrics and Gynecology Hospital, Capital Medical University, Beijing Maternal and Child Health Care Hospital, Beijing 100006, China; 2 School of Nursing, Capital Medical University, Beijing 100069, China

**Keywords:** virtual reality, pelvic floor, Kano model, user demand analysis, serious games

## Abstract

**Highlights:**

**What are the main findings?**

- This study categorized 20 user demands for a VR-based pelvic floor training system into specific Kano attributes.
- Personalization features were identified as primary satisfaction drivers, while exercise guidance and data accuracy represent baseline requirements.

**What are the implications of the main findings?**

- VR may offer theoretical advantages through ‘spatial biofeedback’, potentially enhancing proprioceptive awareness during pelvic floor training.
- Integrating serious game principles into VR rehabilitation tools may support user motivation and anticipated training adherence.

**Abstract:**

**Background:** Poor adherence and monotony in home-based pelvic floor muscle training (PFMT) often lead to suboptimal rehabilitation outcomes. Serious games using virtual reality (VR) may improve training motivation and precision. This study aimed to explore user demands for a VR pelvic floor rehabilitation training system with game-based features. **Methods:** A Kano model-based questionnaire was developed and distributed to patients receiving PFMT. The survey assessed 20 demand items spanning five dimensions: system operation, exercise guidance, personalization, device use, and interaction. Traditional Kano categorization and an optimized mixed-method classification were used to identify core demand attributes. Satisfaction and dissatisfaction indices were also calculated. **Results:** A total of 112 valid questionnaires were analyzed. Using the Kano model, 20 demand items were classified as attractive (*n* = 7), one-dimensional (*n* = 5), must-be (*n* = 6), or indifferent (*n* = 2). Personalization-related demands were mainly identified as attractive attributes, whereas exercise guidance-related demands were primarily classified as must-be or one-dimensional attributes. Satisfaction Index (SI) values ranged from 0.27 to 0.64, and absolute Dissatisfaction Index (DSI) values ranged from 0.34 to 0.71. Optimized Kano analysis identified nine mixed attributes. The questionnaire demonstrated excellent internal consistency (Cronbach’s α = 0.96). **Conclusions:** Participants demonstrated positive willingness to adopt a game-based VR system for PFMT, with diverse needs identified across functional and motivational dimensions. These findings suggest that integrating immersive, personalized, and gamified design features may hold promise for enhancing user engagement and anticipated training adherence, though direct evaluation of clinical effectiveness awaits future prototype-based studies. The identified demand priorities provide structured, evidence-informed guidance for the user-centered design of serious game–oriented VR pelvic floor rehabilitation systems.

## 1. Introduction

Pelvic floor dysfunction (PFD) refers to a group of conditions caused by impairment, degeneration, or weakening of the pelvic support structures, leading to abnormalities in pelvic anatomy and function. Common manifestations include stress urinary incontinence, pelvic organ prolapse, and related reproductive system disorders [1]. If not appropriately managed, PFD may result in irreversible functional impairment. Women with PFD face significantly elevated risks of depression and anxiety, with pooled prevalence of depressive symptoms exceeding 30% [2]. These psychological burdens, compounded by the chronic and often stigmatized nature of symptoms such as urinary incontinence and pelvic organ prolapse, substantially reduce health-related quality of life and social participation [2,3].

Pelvic floor muscle training (PFMT), commonly known as Kegel exercises, is widely recognized as a first-line strategy for the prevention and management of postpartum PFD [4,5]. Despite its proven effectiveness, PFMT requires long-term adherence and correct execution to achieve optimal outcomes. In practice, sustained adherence remains low, particularly in home-based training settings [6,7]. Many individuals experience difficulty accurately identifying and activating pelvic floor muscles, and often inadvertently recruit surrounding muscle groups such as the abdominal or gluteal muscles, which compromises training effectiveness [8]. Moreover, the lack of professional supervision and real-time feedback during unsupervised training may reduce users’ confidence in performing exercises correctly, further undermining motivation and long-term compliance [9].

Virtual reality (VR) technology has attracted growing interest in rehabilitation and exergaming contexts due to its capacity to enhance engagement, motivation, and motor learning through immersive interaction and real-time feedback [10,11,12]. Importantly, the value of VR in rehabilitation does not lie in directly visualizing internal anatomical structures, but in providing an interactive interface that integrates feedback, behavioral cues, and gamified elements to support exercise adherence and self-efficacy [13,14]. Previous studies have demonstrated that VR-based interventions can improve training participation and perceived effectiveness across a range of rehabilitation domains [15,16], suggesting potential applicability to PFMT. The integration of VR in this context is theoretically supported by Self-Determination Theory (SDT), which posits that gamified elements can enhance intrinsic motivation by fulfilling users’ basic psychological needs for autonomy and competence [17]. Furthermore, VR may offer advantages through its capacity to provide visualized biofeedback, potentially allowing users to perceive otherwise imperceptible internal physiological processes through immersive 3D spatial metaphors, which may help address the proprioceptive challenges inherent in traditional PFMT [18].

Before developing or implementing a VR-based pelvic floor rehabilitation training system, it is essential to understand users’ anticipated needs, perceived value, and priority expectations to ensure alignment between system functions and real-world rehabilitation demands. According to the System Development Life Cycle (SDLC), demand analysis is a critical early-stage step that informs system design and resource allocation [19]. The Kano model provides a structured framework for categorizing user requirements based on their asymmetric impact on satisfaction and dissatisfaction, and is particularly suitable for pre-development stages where a functional prototype is not yet available [20]. Unlike technology acceptance frameworks such as the Technology Acceptance Model (TAM), which require participants to have prior exposure to a product, the Kano model enables demand classification based on users’ anticipated needs and expectations, making it well-suited to the conceptual design stage of system development [21]. Furthermore, the Kano model supports design prioritization by distinguishing between features that drive satisfaction and those that prevent dissatisfaction—a distinction not captured by simple importance rankings. This approach has been increasingly applied in digital health and rehabilitation system design contexts [22,23].

Despite growing interest in VR-based rehabilitation, no published study has specifically examined anticipated user demand priorities for a VR pelvic floor rehabilitation system from a pre-development, user-centered demand analysis perspective. Existing studies [24,25] have primarily focused on post-implementation usability or clinical outcomes, leaving a gap in structured needs assessment at the conceptual design stage. This study therefore addresses the following research question: What are the types and priorities of anticipated user demands for a conceptual VR-based PFMT system as classified by the Kano model, and how are these demands distributed across functional dimensions?

This study therefore aims to conduct a user-centered demand analysis of a conceptual VR-based pelvic floor rehabilitation training system using the Kano model, with the goal of providing evidence-informed guidance for subsequent system design and development. The remainder of this paper is organized as follows. The Materials and Methods section describes participant recruitment, questionnaire development, and statistical procedures. The Results section presents demographic characteristics, Kano attribute classifications, SI/DSI analysis, mixed-category findings, and subgroup analyses of VR adoption willingness. The Discussion interprets these findings in relation to VR rehabilitation design and user motivation theory, followed by a Practical Implications section and Strengths and Limitations.

## 2. Materials and Methods

### 2.1. Participants

Participants were recruited from the Female Pelvic Floor Dysfunction Diagnosis and Treatment Center of Beijing Obstetrics and Gynecology Hospital between April 2024 and September 2024. This study was approved by the Medical Ethics Committee of Beijing Obstetrics and Gynecology Hospital, approval number 2024-KY-095-01.

Inclusion criteria were as follows: (1) individuals who were currently undergoing, planning to undergo, or had previously completed pelvic floor rehabilitation training; (2) those with clear consciousness and normal communication abilities, and who were able to express their views independently; and (3) individuals who provided informed consent and voluntarily agreed to participate.

Exclusion criteria included: (1) a history of pelvic surgery, severe urological disorders, or musculoskeletal injuries that could interfere with participation; (2) diagnosed serious cardiovascular, renal, or psychiatric conditions; and (3) medical limitations preventing the safe use of VR equipment. All participants were informed of their right to withdraw from the study at any time without any consequences.

Sample size estimation was conducted using Kendall’s method [26], which recommends recruiting 5–10 participants per independent variable. Given the 20 demand attributes included in this study, a sample size of 120–210 participants was considered appropriate, with an additional allowance of up to 20% attrition. Participant recruitment and eligibility screening were conducted by trained research nurses affiliated with the hospital, who verified inclusion and exclusion criteria and obtained written informed consent prior to questionnaire administration. Ultimately, 146 participants were recruited, satisfying the recommended sample size requirements.

The final analyzed sample of 112 valid questionnaires falls slightly below the lower bound of the pre-estimated range (120–210 participants). While this may modestly limit the statistical stability of Kano classifications for items with marginal category boundaries, the sample nonetheless satisfies the minimum threshold of five participants per variable recommended by Kendall’s method for the 20 demand attributes included in this study.

### 2.2. Survey Questionnaire

The questionnaire consisted of two sections. The first section collected demographic and background information, including age, education level, occupation, experience with pelvic floor rehabilitation, exercise frequency, satisfaction with existing rehabilitation tools, perceived difficulties in performing pelvic floor exercises, and willingness to adopt VR-based rehabilitation approaches.

The second section was a self-developed demand questionnaire based on the Kano model. It was designed to assess users’ anticipated needs and expectations regarding a conceptual VR-based pelvic floor rehabilitation training system, rather than to evaluate actual user experience with an implemented product. The questionnaire comprised 20 paired items across five dimensions: system operation (4 items), exercise guidance (5 items), personalization (3 items), device usage (4 items), and interaction features (3 items).

Each demand item included a pair of functional (positive) and dysfunctional (negative) descriptions reflecting the presence or absence of a specific system attribute. For example:

*1A: “The VR pelvic floor rehabilitation training system provides beginner guidance, user manuals, and tutorial videos. How would you feel?”*

*1B: “If not, how would you feel?”*

The questionnaire was administered in Chinese, which is the participants’ native language. All items were developed and reviewed in Chinese to minimize potential language-related misunderstanding. Participants rated their responses using the standard Kano evaluation scale: “I like it that way,” “It must be that way,” “I am neutral,” “I can live with it that way,” and “I dislike it that way.” Based on the combination of responses to each functional–dysfunctional pair, demand attributes were classified into six Kano categories: Must-be (M), One-dimensional (O), Attractive (A), Indifferent (I), Reverse (R), and Questionable (Q).

The 20 demand items were identified through a multi-step development process. First, a preliminary pool of demand attributes was generated based on a literature review of PMFT, VR–based rehabilitation systems, and serious game design principles. Second, discussions were conducted within the research team to refine and consolidate overlapping items. Third, an expert panel review was performed to evaluate the relevance, clarity, and clinical applicability of each item. The expert panel consisted of 3 senior pelvic floor rehabilitation clinicians, 2 nursing informatics experts, and 2 rehabilitation engineers with experience in VR system design. Content validity was established through the expert panel review: each demand item was independently assessed for relevance, clarity, and clinical applicability by all seven members, and items achieving majority consensus were retained. Wording refinements were subsequently made based on panel feedback to ensure alignment with clinical practice and user-centered design objectives. The overall item-level content validity index (I-CVI) was 0.86. The questionnaire was also reviewed for face validity by researchers prior to full distribution, and minor wording refinements were made based on their feedback.

Internal consistency reliability was assessed using the same dataset included in the final analysis (N = 112). The overall Cronbach’s alpha coefficient of the demand questionnaire was 0.96, and the split-half reliability analysis yielded a reliability coefficient of 0.87, indicating internal consistency. To address potential response bias, the Kano model’s inherent bipolar (functional and dysfunctional) question structure was utilized as a built-in logical check. Responses that were logically inconsistent (categorized as ‘Questionable’) were monitored to ensure the validity of the survey data.

### 2.3. Investigation Methods

To quantify the influence of each demand attribute on user satisfaction, the Satisfaction Index (SI) and Dissatisfaction Index (DSI) were calculated according to established Kano analysis methods [27]. The indices were computed as follows: SI = $\frac{A\;+\;O}{A\;+\;O\;+\;M\;+\;I}$ and DSI = $\frac{O\;+\;M}{A\;+\;O\;+\;M\;+\;I}$. The SI reflects the extent to which fulfilling a given demand contributes to increased satisfaction, whereas the DSI represents the degree of dissatisfaction that may arise if the demand is not met. The absolute values of SI and DSI range from 0 to 1, with higher values indicating a stronger influence on satisfaction or dissatisfaction, respectively [20].

A two-dimensional Kano matrix was constructed by plotting SI values on the vertical axis and absolute DSI values on the horizontal axis. The mean SI and mean absolute DSI values were used as reference thresholds to divide the matrix into four quadrants, corresponding to Attractive, One-dimensional, Must-be, and Indifferent attributes. This relative quadrant analysis enabled prioritization of demand attributes during the early design stage of system development.

To address limitations of the traditional Kano classification—which relies solely on the highest frequency category—we further applied an optimized mixed-category classification method [28,29]. This approach incorporates Total Strength (TS) and Category Strength (CS) to capture the intensity and distribution of user expectations for each demand attribute. These indices were calculated as follows: TS = $\frac{M\;+\;A\;+\;O}{A\;+\;O\;+\;M\;+\;I\;+\;R\;+\;Q}$ and CS = $\frac{\max\{M,A,O,I,Q,R\}\;-\;\mathrm{second}\;\max\;\{M,A,O,I,Q,R\}}{A\;+\;O\;+\;M\;+\;I\;+\;R\;+\;Q}$ TS reflects the overall intensity of user concern for a given demand item, while CS indicates the degree of response concentration within a single dominant category relative to the remaining categories. When TS ≥ 60% and CS ≤ 6%, the demand attribute was classified as a mixed attribute, indicating heterogeneous user expectations. This approach provides a more nuanced basis for prioritizing design features during the pre-development phase of system design.

### 2.4. Statistical Analysis

All statistical analyses were performed using IBM SPSS Statistics version 26.0 (IBM Corp., Armonk, NY, USA). Descriptive statistics, including frequencies and percentages, were used to summarize demographic characteristics. Demand attributes were classified using both traditional Kano analysis and the optimized mixed-category method. SI and DSI values were calculated to further quantify anticipated user responses and support prioritization of system features. Chi-square tests were conducted to examine associations between participant demographic characteristics (age group and education level) and VR adoption willingness. Statistical significance was set at *p* < 0.05.

## 3. Results

### 3.1. Demographic Characteristics of the Participants

Of the 146 collected questionnaires, 34 were excluded due to incomplete responses or inconsistent answers to paired functional–dysfunctional items. Inconsistencies were identified using the Kano model’s inherent logical validity check: questionnaires in which respondents provided simultaneously positive responses to both the functional and dysfunctional versions of multiple items (categorized as Questionable, Q) were flagged as internally inconsistent; questionnaires with missing responses to one or more paired items were also excluded. As demographic information was collected at intake for all 146 participants, a preliminary comparison of available data suggested no systematic differences in age distribution or education level between excluded and included participants.

**Figure 1:**
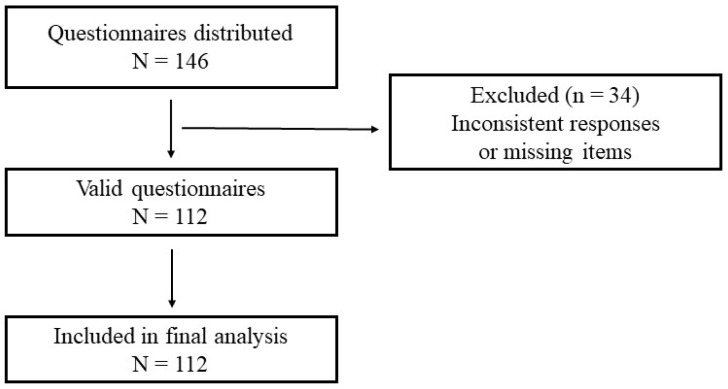
Participant flow diagram.

After excluding invalid responses, a total of 112 valid questionnaires were included in the final analysis. The demographic characteristics of the participants are presented in Table 1. The majority of participants held a bachelor’s degree or above (88.4%). Regarding pelvic floor rehabilitation exercise frequency, 55 out of 112 (49.1%) participants performed pelvic floor rehabilitation exercises 0–4 times per month, while 42 out of 112 (37.5%) participants reported engaging in the exercises 4–8 times per month. Notably, all participants reported at least some degree of willingness to use a VR-based pelvic floor rehabilitation training system (Figure 1).

### 3.2. Subgroup Analysis of VR Adoption Willingness

To assess whether VR adoption willingness varied across participant subgroups, chi-square tests were conducted to evaluate associations between demographic characteristics (age group and education level) and willingness to adopt VR-based rehabilitation. Age was dichotomized into two groups (≤35 years and >35 years), and education was categorized into three groups (junior college or below, bachelor’s degree, and master’s degree or above). VR adoption willingness was classified as high (scores 4–5) versus non-high (scores 1–3). Results indicated no statistically significant difference in high VR adoption willingness (scores 4–5) between age groups (≤35 years: 40.8%; >35 years: 58.5%; χ^2^ = 2.592, df = 1, *p* = 0.107). Similarly, no significant association was found between education level and VR adoption willingness (χ^2^ = 1.538, df = 2, *p* = 0.463), with high willingness reported by 61.5% of participants with junior college education or below, 48.1% with a bachelor’s degree, and 42.2% with a master’s degree or above. These findings suggest that positive attitudes toward VR-based PFMT were relatively consistent across demographic subgroups within this sample, regardless of age or educational background.

### 3.3. Kano Attribute Classification of VR Training System Demands

Based on the traditional Kano evaluation method, the distribution of demand attributes for each item was determined. Among the 20 demand items, 7 were classified as Attractive attributes, 5 as One-dimensional attributes, 6 as Must-be attributes, and 2 as Indifferent attributes (Table 2). No items were classified as Reverse (R) or Questionable (Q).

At the dimensional level, demands related to personalization were predominantly classified as Attractive attributes, whereas most exercise guidance–related items were classified as Must-be or One-dimensional attributes. Items related to device usage and interaction requirements showed a more heterogeneous distribution across Kano categories.

### 3.4. SI and DSI Analysis

SI and DSI were calculated for all 20 demand items. SI values ranged from 0.27 to 0.64, while the absolute values of DSI ranged from 0.34 to 0.71 (Table 2). The mean SI and mean absolute DSI values were 0.46 and 0.54, respectively.

**Figure 2:**
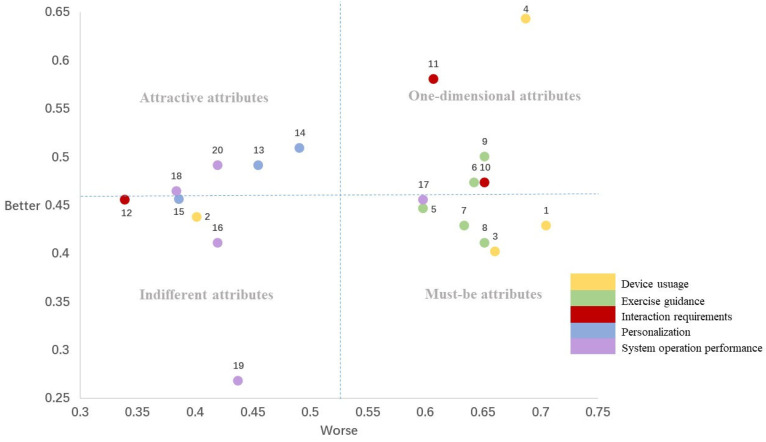
Matrix diagram of VR pelvic floor rehabilitation training system demand. Note: the quadrants were defined using the mean SI and mean absolute DSI values. The numbers (1–20) correspond to the demand items listed in Table 2.

Using these mean values as reference thresholds, a four-quadrant matrix was constructed to visualize and relatively prioritize demand attributes (Figure 2). The quadrant analysis identified 4 Attractive attributes, 5 One-dimensional attributes, 6 Must-be attributes, and 5 Indifferent attributes. As the quadrant boundaries were defined in a relative manner, each attribute was evaluated in comparison with the others. Within the same functional category, attributes with higher SI values and lower absolute DSI values were considered higher priorities for system design optimization, as such attributes are more likely to enhance user satisfaction while minimizing dissatisfaction if unmet, consistent with established applications of the Kano model in system and service design [30].

### 3.5. Mixed Category Analysis Using TS and CS

To further refine demand prioritization, an optimized Kano reclassification was conducted using TS and CS. As shown in Table 3, nine demand items met the criteria for mixed attributes (TS ≥ 60% and CS ≤ 6%).

Specifically, three items related to system durability and environmental personalization (Items 2, 13, and 14) were classified as combined Attractive and Must-be attributes, indicating that while they currently enhance satisfaction, they may evolve into baseline expectations as VR rehabilitation becomes more widely adopted.

Three exercise guidance and system operation items (Items 5, 6, and 17) were identified as mixed Must-be and One-dimensional attributes, reflecting strong but directionally divided user expectations. Two interaction and personalization items (Items 12 and 15) were classified as mixed Attractive and Indifferent attributes, suggesting appeal to specific user subgroups rather than the overall population. Item 16 (novice guidance and tutorials) was identified as a mixed Must-be and Indifferent attribute, indicating heterogeneous expectations regarding onboarding support.

## 4. Discussion

### 4.1. Principal Findings

This study identified that personalization-related features were predominantly classified as Attractive attributes, exercise guidance features as Must-be or One-dimensional attributes, and nine demand items exhibited mixed-category characteristics reflecting heterogeneous user expectations. Subgroup analysis further revealed no significant differences in VR adoption willingness across age or education groups, suggesting broadly consistent user receptiveness within this sample. Taken together, these findings provide practical, user-centered evidence for prioritizing design features in VR-based PFMT systems at the pre-development stage.

All items within the personalization dimension were classified as Attractive attributes, indicating that customizable features—such as visual adjustments, scene selection, and individualized training parameters—have the potential to substantially enhance user satisfaction. Although these features are not essential prerequisites for system acceptance, they serve as important motivational factors that may promote user engagement and sustained participation. Similarly, several interaction-related features, including virtual rewards and simulated communication, were also identified as Attractive attributes. These findings suggest that integrating game-based elements—such as avatars, feedback mechanisms, and immersive environments—may hold promise for enhancing anticipated user experience in PFMT, consistent with evidence from analogous VR rehabilitation contexts [13,14,31].

### 4.2. Theoretical Considerations for VR-Based PFMT

Unlike traditional 2D media-based exercises, VR may offer particular advantages through what has been theorized as ‘spatial biofeedback’—whereby immersive 3D environments may facilitate proprioceptive awareness of pelvic floor muscle activation through visual–motor correspondence [18]. Although participants in this study did not interact with an actual VR system, the observed high demand for immersive interaction features is theoretically consistent with this framework. Known practical challenges of VR devices—including cybersickness, device weight, visual fatigue, and the learning curve associated with head-mounted displays—were beyond the scope of this pre-prototype study and warrant systematic evaluation in future usability research [32].

The three A + M attributes related to system durability and environmental personalization currently function as satisfaction enhancers; however, as VR-based rehabilitation becomes more widely adopted, these features may gradually transition into baseline user expectations. Early prioritization of these features in the design process may therefore help sustain long-term user satisfaction and system competitiveness [28].

In contrast, the two A + I attributes reflecting social and aesthetic preferences appear to have limited impact on overall satisfaction at the population level. Accordingly, their inclusion may be considered optional and dependent on available resources or the characteristics of the target user group.

Interestingly, the two Indifferent attributes—both related to system usability scaffolding—suggest that users may perceive basic interface guidance as an implicit standard of contemporary digital health systems rather than an explicit contributor to satisfaction [33]. In other words, while the absence of these features could negatively affect usability, their mere presence is unlikely to meaningfully enhance perceived value or training motivation. This finding highlights a distinction between functional necessity and satisfaction generation, and underscores the importance of allocating design resources toward features that actively drive engagement once basic usability expectations are met.

Must-be attributes, including the provision of a quiet and immersive training environment, comfort during prolonged use, and accurate measurement and feedback, were consistently identified as essential system requirements. Failure to meet these fundamental expectations may result in dissatisfaction and reduced trust in the system [16,34,35,36,37]. In particular, accurate data acquisition and real-time feedback are critical for effective PFMT, especially in home-based settings where professional supervision is limited. These attributes should therefore constitute the foundational design requirements of any VR-based rehabilitation system. Within the SDT framework, must-be features such as accurate real-time feedback and a supportive training environment may serve as prerequisites for satisfying users’ basic psychological need for competence—a recognized antecedent of intrinsic motivation in rehabilitation [17,38,39]. Whether this theoretical linkage holds in the specific context of VR-based PFMT remains to be confirmed through empirical testing with an implemented prototype.

Subgroup analysis revealed no statistically significant differences in VR adoption willingness by age or education level. Although older participants (>35 years) showed a somewhat higher proportion of high willingness (58.5% vs. 40.8%), this difference did not reach statistical significance, likely reflecting the sample’s demographic homogeneity. These findings tentatively suggest that positive attitudes toward VR-based PFMT may be broadly consistent across the patient demographic range examined, which could support the feasibility of general clinical implementation without requiring highly targeted demographic segmentation in initial rollout phases [40]. However, given the sample’s educational and age profile, these results should be interpreted cautiously and replicated in more demographically diverse populations.

Overall, the findings highlight the potential of applying serious game principles to VR pelvic floor rehabilitation system design. By strategically integrating Attractive and One-dimensional attributes—such as progress visualization, personalized feedback, reward mechanisms, and immersive interaction—developers may aim to support intrinsic motivation and anticipated training adherence. The combination of immersive environments, biofeedback, and personalization may potentially strengthen users’ sense of control and engagement, which are recognized determinants of adherence in rehabilitation interventions [41,42]. However, as this study assessed anticipated user demands rather than direct system interaction, whether these design features will translate into improved adherence and clinical outcomes in practice requires validation through future prototype-based usability and effectiveness studies.

## 5. Practical Implications

These findings carry several implications for the design and development of VR-based pelvic floor rehabilitation systems. Personalization features (Attractive attributes) should be prioritized as satisfaction differentiators supporting long-term engagement, while must-be attributes—accurate feedback, wear comfort, and immersive environment—constitute non-negotiable design foundations.

Among the nine mixed-category attributes, A + M attributes (performance stability, personalized settings, adjustable environments) are likely to become baseline expectations as VR rehabilitation matures and should be addressed early. A + I attributes (social interaction, color customization) may be deferred to later iterations based on available resources. The I + M classification of novice guidance suggests adaptive onboarding designs that can be bypassed by experienced users. The consistent VR adoption willingness across demographic subgroups supports general clinical implementation without requiring demographic-specific adaptations at the outset.

## 6. Strengths and Limitations

This study has several strengths. The dual-method Kano approach combining traditional classification with optimized TS/CS-based mixed-category analysis provided more nuanced demand insights than either method alone. The questionnaire underwent rigorous multi-step development including literature review and expert panel review by seven specialists across clinical, nursing informatics, and rehabilitation engineering domains. The study addresses a clinically relevant gap by providing structured, user-centered demand evidence at the pre-prototype design stage.

Several limitations should be acknowledged. First, the sample was predominantly young and highly educated, limiting generalizability to older or less digitally literate populations. Second, prior VR experience was not recorded, which may have influenced feature prioritization. Third, exclusion of 34 questionnaires due to inconsistent responses may introduce selection bias that could not be fully assessed. Fourth, as participants had no direct VR interaction, this study captured anticipated rather than post-use demands; alignment with actual usability and clinical effectiveness requires prototype-based validation. Fifth, hardware-related concerns including cybersickness, device weight, and visual fatigue were not assessable in this pre-prototype context and should be evaluated in future usability studies. Sixth, the non-significant subgroup findings (*p* > 0.05) may partly reflect limited statistical power due to sample homogeneity; future studies with more diverse populations should examine whether demographic factors moderate demand priorities.

## 7. Conclusions

This study identified and prioritized anticipated user demands for a conceptual VR-based pelvic floor rehabilitation training system using the Kano model. Personalization features were classified as Attractive attributes, exercise guidance and data accuracy as Must-be or One-dimensional attributes, and nine items exhibited mixed-category characteristics reflecting heterogeneous user expectations. Subgroup analysis revealed no significant differences in VR adoption willingness by age or education level, suggesting broadly consistent user receptiveness within this sample.

These findings offer structured, evidence-informed guidance for the user-centered design of serious game-oriented VR rehabilitation systems at the pre-development stage. Future work should progress to prototype-based usability testing and clinical effectiveness evaluation to validate and extend these demand-level insights.

## Figures and Tables

**Table 1:** Basic characteristics of participants (N = 112).

Category	N (%)
**Age (years)**	
≤25	1 (0.9)
25–35	70 (62.5)
35–45	33 (29.5)
45–55	7 (6.3)
>55	1 (0.9)
**Education**	
Senior high school and below	4 (3.6)
Junior college	9 (8.0)
Bachelor’s degree	54 (48.2)
Master’s degree and above	45 (40.2)
**Occupation**	
Students	1 (0.9)
Laborers	1 (0.9)
Company employees	53 (47.3)
Public institutions employees	35 (31.3)
Freelancers	11 (9.8)
Others	11 (9.8)
**Frequency of pelvic floor rehabilitation exercises**	
0–4 times/month	55 (49.1)
4–8 times/month	42 (37.5)
8–12 times/month	10 (8.9)
12 times and above/month	5 (4.5)
**Willingness to use VR pelvic floor rehabilitation training device**	
Not needed	0 (0.00)
Interested	52 (46.4)
Not important	7 (6.3)
Needed	40 (35.7)
Very needed	13 (11.6)

**Table 2:** Kano attributes of demand for VR pelvic floor rehabilitation training system.

Dimension		Item	Category Totals				Traditional Category	SI	DSI
			**A**	**O**	**M**	**I**			
**Device usage**	1	Relaxing training environment	19	29	50	14	M	0.43	−0.71
	2	Long-term performance stability	36	13	32	31	A	0.44	−0.40
	3	Device wearing comfort	23	22	52	15	M	0.40	−0.66
	4	Mainstream VR device compatibility	23	49	28	12	O	0.64	−0.69
**Exercise guidance**	5	Measurement data accuracy	15	35	32	30	O	0.45	−0.60
	6	Real-time movement feedback	14	39	33	26	O	0.47	−0.64
	7	Varied training modes	24	24	47	17	M	0.43	−0.63
	8	Individualized training plan customization	26	20	53	13	M	0.41	−0.65
	9	Historical data and progress reports	25	31	42	14	M	0.50	−0.65
**Interaction requirements**	10	Virtual character interaction	23	30	43	16	M	0.47	−0.65
	11	Virtual reward system	25	40	28	19	O	0.58	−0.61
	12	Social interaction simulation	38	13	25	36	A	0.46	−0.34
**Personalization**	13	Personalized display and training settings	34	21	30	27	A	0.49	−0.46
	14	Adjustable virtual environment	31	26	29	26	A	0.51	−0.49
	15	Color customization options	36	16	28	34	A	0.46	−0.39
**System operation performance**	16	Novice guidance and tutorials	30	16	31	35	I	0.41	−0.42
	17	Voice guidance and multilingual support	17	34	33	28	O	0.46	−0.60
	18	Regular software updates	39	13	30	30	A	0.46	−0.38
	19	Simple and intuitive interface	17	13	36	46	I	0.27	−0.44
	20	Stable lag-free operation	36	19	28	29	A	0.49	−0.42

Note: A is the attractive attribute; I is the indifferent attribute; M is the must-be attribute; O is the one-dimensional attribute. Full item descriptions: Item 1: Provides a relaxing and quiet virtual training environment; Item 2: Ensures long-term use without performance degradation; Item 3: Designed for comfort, allowing extended wear without discomfort; Item 4: Compatible with various mainstream VR devices, including headsets, controllers, and more, offering flexibility for users to choose based on their needs; Item 5: Ensures the accuracy of all measurements and feedback data; Item 6: Provides real-time feedback to accurately guide muscle movement and power output; Item 7: Offers a variety of training modes, covering aspects such as intensity and endurance; Item 8: Allows users and medical professionals to customize individualized training plans; Item 9: Records and analyzes the user’s historical training data, generates summary reports with professional analysis, and offers optimized future training plans; Item 10: Enables interaction with virtual characters for situational reflex training and similar activities; Item 11: Provides virtual rewards to motivate users during training; Item 12: Simulates realistic communication and interaction with other users; Item 13: Personalizes settings, including display preferences, visual adjustments, and training parameters; Item 14: Adjusts the virtual environment according to users’ preferences, such as scenes and music; Item 15: Offers a rich array of color customization options; Item 16: Provides novice guidance, user manuals, and tutorial videos; Item 17: Includes voice guidance options and supports multiple languages; Item 18: Delivers regular software updates and maintenance; Item 19: Features a simple, easy-to-use interface; Item 20: Operates stably without lag.

**Table 3:** Mixed category of demand of VR pelvic floor rehabilitation training device.

Items	Traditional Category	TS	CS	Mixed Category
1	M	0.88	0.19	M
2	A	0.72	0.04	H (A + M)
3	M	0.87	0.26	M
4	O	0.89	0.19	O
5	O	0.73	0.03	H (O + M)
6	O	0.77	0.05	H (O + M)
7	M	0.85	0.21	M
8	M	0.88	0.24	M
9	M	0.88	0.10	M
10	M	0.86	0.12	M
11	O	0.83	0.11	O
12	A	0.68	0.02	H (A + I)
13	A	0.76	0.04	H (A + M)
14	A	0.77	0.02	H (A + M)
15	A	0.70	0.02	H (A + I)
16	I	0.69	0.04	H (I + M)
17	O	0.75	0.01	H (O + M)
18	A	0.73	0.08	A
19	I	0.59	0.09	I
20	A	0.74	0.06	A

Note: A is the attractive attribute; I is the indifferent attribute; M is the must-be attribute; O is the one-dimensional attribute; H is the mixed attribute (the attributes being mixed are specified in parentheses).

## Data Availability

The raw data supporting the conclusions of this article will be made available by the authors on request due to privacy restrictions.
